# Internet-based cognitive behavioral therapy for anxiety and depressive symptoms in Brazilian emerging adults: A pilot randomized controlled trial^☆^

**DOI:** 10.1016/j.invent.2025.100854

**Published:** 2025-07-09

**Authors:** Juliana Maltoni, Carmem Beatriz Neufeld, Victoria Aminoff, Gerhard Andersson

**Affiliations:** a Faculty of Philosophy, Sciences and Letters at Ribeirão Preto, Department of Psychology, University of São Paulo, Av. Bandeirantes, 3900, Ribeirão Preto, SP 14040-900, Brazil; b Department of Behavioural Sciences and Learning, Linköping University, SE-581 83 Linköping, Sweden; c Department of Biomedical and Clinical Sciences, Linköping University, SE-581 83 Linköping, Sweden; d Department of Clinical Neuroscience, Karolinska Institute, 171 77 Stockholm, Sweden

**Keywords:** Internet-based cognitive-behavioral therapy, Anxiety, Depression, Young adults, Randomized controlled trial

## Abstract

**Background:**

Anxiety and depressive disorders are highly prevalent in Brazil, with higher vulnerability among young adults. Despite the high prevalence, Brazil faces significant challenges in its mental health care system, with only a minority receiving treatment. Tailored internet-delivered cognitive behavioral therapy (ICBT) offers a promising strategy to address this treatment versus demand gap. This study examines the efficacy of individually tailored ICBT intervention with on-demand support for reducing anxiety and depressive symptoms in young adults.

**Methods:**

This two-arm randomized controlled trial involved Brazilian young adults (aged 18–24 years) who were randomly assigned to either a treatment group (*n* = 46) or a waitlist control group (*n* = 46). The 8-week treatment included individually tailored ICBT with therapist support on-demand via chat, conducted on an online platform. Primary outcomes were symptoms of anxiety and depression. Secondary measures included stress, insomnia, smartphone and social media use, perfectionism, and quality of life. A six-month follow-up was conducted.

**Results:**

Multiple regression analysis indicated that the treatment group, in comparison to the control group, showed significant reductions in anxiety, depression, stress, and insomnia, as well as improvements in quality of life, with moderate to large effects sizes.

**Conclusion:**

ICBT is a viable intervention for young Brazilians experiencing common mental health symptoms. Further research is needed to explore implementation and impact on other populations.

## Highlights

• This randomized controlled trial investigated a tailored ICBT for young Brazilians
• ICBT was effective in reducing anxiety and depressive symptoms
• Large effect sizes were found for depression, stress, and insomnia symptoms
• Moderate effect sizes were found for anxiety and improvements in quality of life

## 1 Introduction

Mood and anxiety disorders are highly prevalent in the general population, with Brazil ranking among the countries with the highest rates—9.3 % for anxiety and 5.8 % for depressive symptoms (World Health Organization, 2017). These disorders often begin early in life, with approximately 75 % of lifetime cases emerging by age 24 (Kessler et al., 2005), emphasizing the need for early intervention. This age group shows heightened vulnerability and psychological distress compared to other age groups (Lopes et al., 2022; Varma et al., 2021). In Brazil, the prevalence of depression nearly doubled among young adults aged 18 to 24, rising from 5.6 % in 2013 to 11.1 % in 2019. Socioeconomic factors could play a role in those results, since this subgroup is one of the most vulnerable for economic crises experienced in those years (Lopes et al., 2022).

Mental health care system in Brazil developed through the years a notable example of psychiatric reform, resulting in the establishment of a community-oriented mental health network that provides universal access to mental health services (Marchionatti et al., 2023). However, despite successes, Brazil's national mental health care system faces significant challenges, including low funding, a high proportion of patients in specialized services, a shortage of professionals, and uneven service coverage (Amaral et al., 2018).

The prevalence rates of anxiety and major depression reach 20.9 % and 12.6 %, respectively, among Brazilian young adults, with a comorbidity rate of 9.7 %, and strong associations with lower education, socioeconomic status, and unemployment (Molina et al., 2014). Depression and anxiety in youth are also associated with excessive internet and social media use, poor sleep, high perfectionism, and low self-esteem (Ahmed et al., 2024; Egan et al., 2011; Ramón-Arbués et al., 2020). Therefore, addressing related psychological and behavioral domains is essential for delivering comprehensive and effective treatment, particularly among young adults.

Cognitive-behavioral therapy (CBT) is an established and effective treatment for a variety of disorders, including depression and anxiety (Hofmann et al., 2012). However, WHO World Mental Health (WMH) survey results show that only a minority of people receive evidence-based treatments for common mental disorders, with even lower treatment rates among those with low socioeconomic status. For 12 month DSM-IV/CIDI cases, only 22.0 % of individuals in upper-middle-income countries, like Brazil, received any treatment, compared to 36.8 % in high-income countries (Evans-Lacko et al., 2018).

Over the past 20 years, ICBT has become more common and accessible, offering a promising avenue for increasing access to evidence-based psychological treatments (Andersson et al., 2019b). Clinician-supported ICBT has shown similar effectiveness to face-to-face CBT (Hedman-Lagerlöf et al., 2023) and is cost-effective (Andersson et al., 2019b; Richards et al., 2020), making it a viable tool to bridge the treatment gap and improve access to mental health care.

Considering the comorbidity between these disorders (Johansson et al., 2013), transdiagnostic and tailored ICBT presents a promising strategy for addressing overlapping symptoms across multiple conditions, given that ICBT can effectively address multiple symptoms simultaneously, reach a broader audience, and making them a practical approach for both treatment and prevention. Tailoring these programs to individual needs ensures that they are more personalized and potentially more effective (Andersson and Titov, 2014; Păsărelu et al., 2017). Those strategies in ICBT programs have been effective in treating various psychiatric and somatic conditions (van Beugen et al., 2014; Hennemann et al., 2022), such as depression and anxiety (Dear et al., 2018; Richards et al., 2020; Salamanca-Sanabria et al., 2020). Specifically for young people, ICBT has shown promising results in reducing symptoms of anxiety and depression (Berg et al., 2022; Topooco et al., 2018). Consequently, international health organizations increasingly advocate for internet-based mental health interventions (Pan American Health Organization, 2018; World Health Organization, 2017).

Although there are ICBT initiatives with on-demand support in Latin American settings (Salamanca-Sanabria et al., 2020), only two Brazilian studies have been conducted to our knowledge, addressing sexual health (Fischer et al., 2024) and depression (Lopes et al., 2023). Given the potential of ICBT to reduce the treatment gap in primary care in Brazil and the high prevalence of common mental disorders, making an ICBT program available targeting common mental health symptoms is crucial. Considering that 81 % of the Brazilian population over 10 years old uses the internet, especially via mobile phones, the feasibility of such interventions is possible (Comitê Gestor da Internet no Brasil [CGI], 2020).

### 1.1 Objectives

The first aim of this study was to test the efficacy of individually tailored ICBT with support on demand for reducing anxious and depressive symptoms in young adults aged between 18 and 24 years.

The second aim was to investigate treatment effects on secondary measures of stress, insomnia, perfectionism, quality of life, and problematic Internet use.

## 2 Methods

### 2.1 Study design

The study was a two-armed pilot RCT with participants allocated to either a treatment group or a waitlist control group. Both groups received the same intervention, but the waitlist control group received it after post-treatment measures had been collected. All data collection, treatment administration, and storage were managed through an online treatment platform (Iterapi platform) (Vlaescu et al., 2016), which encrypts and stores all data on a secure server. Due to the nature of the psychological intervention, blinding was not feasible.

This study was approved by the Research Ethics Committee of the Faculty of Philosophy, Sciences and Letters of Ribeirão Preto (CAAE No. 59,445,522.5.0000.5407). Only those participants who consent to participate in the research by signing the consent form were allowed to participate in the research.

### 2.2 Participants and recruitment

Participants were recruited through paid advertisements on Instagram, specifically targeting individuals aged 18–24 years in Brazil. Additionally, WhatsApp, Facebook, and email were also used, employing a snowball sampling approach. The focus of the advertisement was an online intervention for common mental health symptoms such as depression, anxiety, and stress. A link to the study website (www.secuidalapicc.com) was included in the advertisement, offering potential participants detailed information about the intervention and a registration button to initiate enrollment. The registration was the first assessment of participants and included sociodemographic questions and the instruments: Depression, Anxiety and Stress Scale-21 (DASS-21), Insomnia Severity Index (ISI), Almost Perfect Scale-.

Revised (APS-R), Brunnsviken Brief Quality of Life Scale (BBQ), Smartphone Addiction Scale-Short Version (SAS-SV) and an adapted version of the Social Media Disorder Scale-Short Form (SMDS-SF).

To proceed with the assessment, participants signed an electronic consent form and provided an email address. Upon registration, they were assigned a unique access code to the platform. The program's effects were evaluated across four assessments: T1) recruitment (pre-treatment); T2) post-treatment for the treatment group and a second assessment for the control group; T3) post-treatment for the control group; and T4) a follow-up after 6 months of intervention for each group. Instruments were the same for each assessment time for both groups, with additional feedback questions specific to treatment and waitlist experiences based on group assignment. Participants could also evaluate the module content upon completion. A psychotherapist also called the participants during this period to encourage assessment completion and to gather supplemental data. This second assessment period lasted for two weeks.

**Fig. 1:**
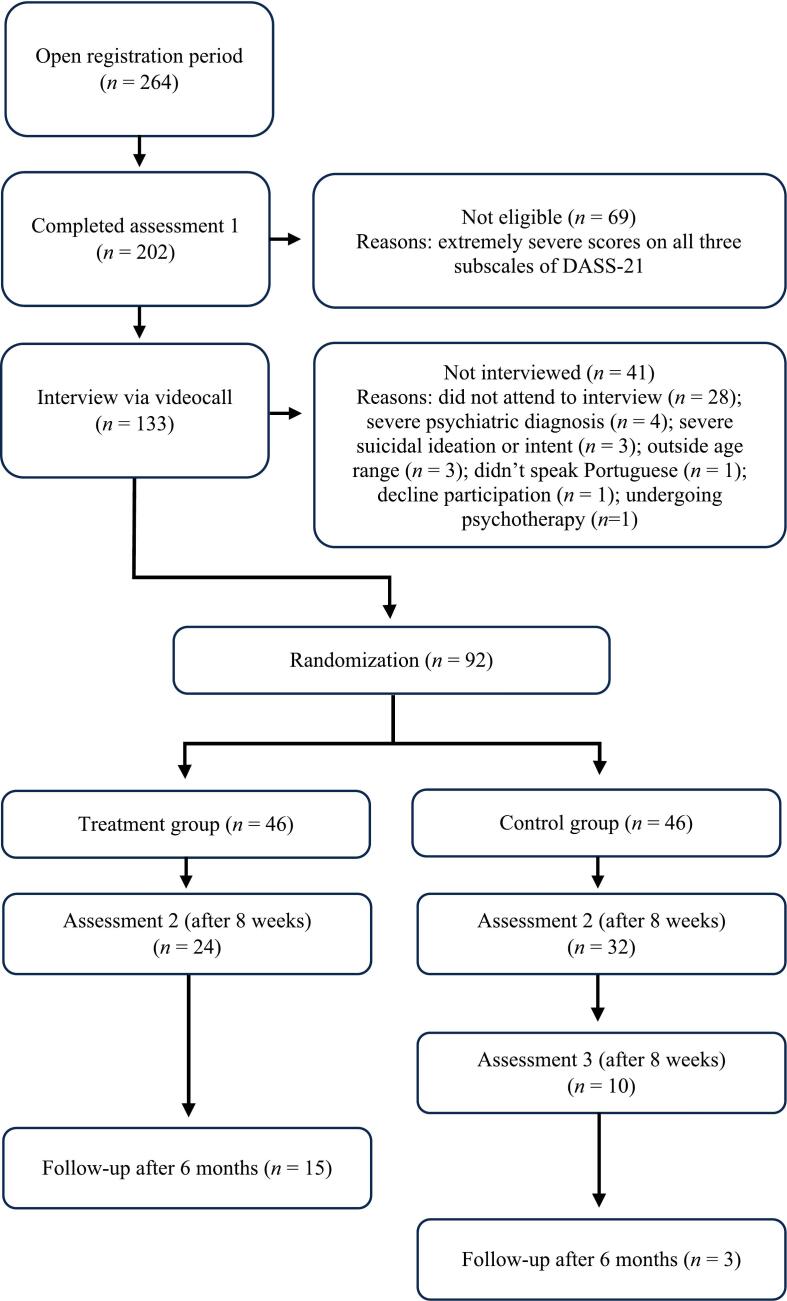
Participant flowchart.

As illustrated in Fig. 1, a total of 92 individuals were included and randomized into either treatment (*n* = 46) or control group (*n* = 46) using the platform's automated allocation function, performed by the lead researcher. Only those who completed initial screening were evaluated for eligibility, and those who met inclusion criteria received a WhatsApp or email invitation to schedule a brief video interview with a psychologist. This interview aimed to confirm symptom severity, screen for additional issues (e.g., substance use, other mental health disorders), assess suicidal ideation, identify relevant areas for intervention tailoring, and explain the intervention in further detail. Individuals who were ineligible were referred to other public mental health services.

By the time that treatment group started, both groups received login credentials for the platform. Only the treatment group had access to the intervention and the messaging channel. During the waiting period, the control group could ask questions about the study via email. After the beginning of intervention, all communication occurred via the platform or e-mail. During all the four moments of the assessments, participants received automatically email reminders. All data collected were stored on a secure server at Linköping University, Sweden.

### 2.3 Eligibility criteria

The inclusion criteria to participate the study was (1) 18 to 24 years old; (2) own a device with Internet access; (3) speak and read Brazilian Portuguese. Individuals were not eligible if they (1) had a severe psychiatric diagnosis (bipolar disorder, schizophrenia or substance abuse); (2) reported severe suicidal ideation or suicidal intent; (3) were in an ongoing psychological and/or psychiatric treatment; (4) had changed psychiatric medication <3 months before the intervention; (5) had extremely severe scores on all three subscales of DASS-21 (28+ for depression, 20+ for anxiety and 34+ for stress) (Lovibond and Lovibond, 1995). Since those scores indicate a high level of psychological distress, participants who didn't meet the criteria were guided toward alternative treatment options of appropriate intensity and focus, as available through other health services.

### 2.4 Treatment

The intervention program, named Se Cuida (“Take Care”), was based on CBT principles focused on the management of anxiety and depression symptoms in young adults. Delivered via a fully responsive online platform optimized for smartphone access, the program's materials included texts, vignettes, videos, and exercises, all available for download at any time during the intervention.

The study involved adapting previous studies for the Brazilian context of previous studies in collaboration with a Swedish institution specializing in online interventions, led by the last author of this project (Aminoff et al., 2023; Berman et al., 2024; Topooco et al., 2018). The adaptation process proposed in this work followed four key steps: selection of materials, initial translation and modification, expert evaluation of the translation, and focus groups with experts and target population. After selecting suitable ICBT programs that had been evaluated in randomized controlled trials, the materials were translated into Portuguese, with adaptations made to fit the Brazilian target audience and study objectives. Two focus groups were conducted: one with experts in psychology (*n* = 4) and one with target population (*n* = 7), to evaluate the translated materials for semantic, idiomatic, and cultural relevance. Their feedback was incorporated into a final version, which was then adapted for the platform interface and visual content.

The intervention was based on CBT principles and aimed to address common psychological symptoms experienced by young people, such as anxiety, depression, stress, procrastination, sleep-related problems, perfectionism, low self-esteem and behaviors associated with problematic internet use. This last module was developed by the principal investigator during this project and was the only one that was not adapted from the original programs. Each module consisted of an average of 16.5 pages (SD = 3) and 4026 words (SD = 606). Table 1 details module content and access rates.

**Table 1:** Content of intervention modules and their access.

Week	Module	Access to modules (*n* = 45) (n) (%)
1	Psychoeducation about psychological distress. Intervention guide and setting goals	40/45 (88 %)
2	Behavioral analysis to identify maladaptive patterns	33/45 (73.3 %)
3⁎	Behavioral activation and coping with procrastination or coping with sleep problems	18/26 (69.23 %) 11/19 (57.89 %)
4	Reduce problematic Internet use	25/45 (55.55 %)
5	Strategies for cognitive restructuring	22/45 (48.88 %)
6⁎	Coping with anxiety symptoms or social anxiety or panic or generalized anxiety	6/12 (50 %) 6/9 (66.66 %) 3/6 (50 %) 7/18 (38.88 %)
7⁎	Coping with stress or perfectionism or self esteem	4/6 (66.66 %) 14/24 (58.23 %) 3/15 (20 %)
8	Relapse prevention and modules review	19/45 (42.22 %)

⁎ Tailored modules.

The treatment lasted for eight weeks and, during which time participants received eight out of the 14 available modules, one per week. Modules 1, 2, 4, 5 and 8 were delivered for all participants, while modules 3, 6, and 7 were tailored according to participants' individual needs evaluated during screening and interviews. Each module had weekly interactive exercises related to its content. Participants could answer and save their exercises, which are accessible to therapists for review. The therapist team was composed of 4 clinical psychologists, including the main author of this study. The psychologists offered weekly feedback and/or encouragement on exercise completion, and the therapist team held weekly discussions to review cases and consult with the study authors as necessary.

Participants in the treatment group had access to a licensed clinical psychologist through the message channel on the platform. Guidance is recognized as a key factor in enhancing treatment adherence in ICBT (Baumeister et al., 2014). Therefore, we incorporated initial assessments, regular encouraging feedback, and reminder phone calls at the conclusion of the intervention (Dziura et al., 2013). Each participant was assigned a specific psychologist available during business hours, allowing participants to reach out for program-related support or assistance as needed. All communication was asynchronous and unscheduled, with responses provided within 24 h. Psychologists offered weekly feedback and encouragement on exercise completion, and the therapist team held weekly discussions to review cases and consult with the study authors as necessary.

Any reports of risk behaviors, suicidal ideation, or symptom exacerbation were carefully monitored by the assigned psychotherapist. Participants who no longer met eligibility criteria during the study were not withdrawn from the intervention; instead, they were guided toward alternative treatment options of appropriate intensity and focus, as available through other health services.

Any risk behaviors, suicidal ideation, or increased symptom severity reported during treatment were evaluated by the psychotherapist in charge of the participant. Participants who no longer met eligibility criteria during the study were not withdrawn from the intervention; instead, they were guided toward alternative treatment options of appropriate intensity and focus, as available through other health services.

## 3 Measures

### 3.1 Primary outcomes

Anxiety and depressive symptoms were considered the primary outcomes and were assessed with the DASS-21 (Lovibond and Lovibond, 1995) in its validated Brazilian form (Vignola and Tucci, 2014). The instrument consists of 3 subscales evaluating anxiety, depression and stress with 21 questions rating symptoms severity from 0 (does not apply to me at all) to 3 (applied to me very much or most of the time). The original authors proposed categories for symptomatology for each subscale (normal, mild, moderate, severe, and extremely severe). The internal consistency for the validated instrument was good for all subscales: depression (0.92), stress (0.90) and anxiety (0.86). Severity rating can be accessed in the work of Vignola and Tucci (2014).

### 3.2 Secondary outcomes

Secondary outcomes included assessments of stress, insomnia, perfectionism, quality of life, and problematic internet and social media use. Stress was measured using the stress subscale of the DASS-21. Insomnia symptoms were assessed with the Insomnia Severity Index (ISI) (Bastien et al., 2001), using a validated version (Castro, 2011), which comprises seven items rated on a scale from 0 to 4. The ISI categorizes scores as follows: non-significant insomnia (0–7), subthreshold insomnia (8–14), moderate clinical insomnia (15–21), and severe clinical insomnia (22–28). The validated instrument reported a good reliability and internal consistency (0.86).

Perfectionism was measured using the Almost Perfect Scale - Revised (APS-R) (Slaney et al., 2001), a 23-item instrument with three subscales (Discrepancy,

Standards, and Order) rated from 1 (strongly disagree) to 7 (strongly agree).

Maladaptive perfectionism is indicated by a combined score of 42 or higher on the Standards and Discrepancy subscales. The version validated by Soares et al. (2020) demonstrated good reliability across dimensions.

Quality of life was assessed using the Brunnsviken Brief Quality of Life Scale (BBQ) with a translated version (Lindner et al., 2016). The BBQ includes 12 items measuring satisfaction and importance across six life domains, with responses ranging from 0 (completely disagree) to 5 (completely agree). Higher scores indicate greater quality of life, ranging from 0 to 96. The BBQ has demonstrated good internal consistency and test-retest reliability, with clinical populations scoring lower on average (38.7) compared to non-clinical populations (60.1).

Problematic smartphone use was assessed with the Smartphone Addiction ScaleShort Version (SAS-SV), using a validated Portuguese version (Andrade et al., 2020) adapted from Kwon et al. (2013). This scale contains 33 items rated from 1 (strongly disagree) to 6 (strongly agree), with cutoff scores of 31 for men and 33 for women. Internal consistency for this scale was 0.81.

Problematic social media use was evaluated using the Social Media Disorder.

Scale-Short Form (SMDS-SF) (Van Den Eijnden et al., 2016), which aligns with DSM5 criteria for internet dependence disorder. The SMDS-SF includes nine dichotomous items (yes = 1, no = 0), with a cutoff score of 5 indicating problematic use. For this study, we adapted Costa et al. (2016)’s Portuguese version for young adults in Brazil, adding minor language adjustments and four supplementary questions about social media habits, including daily usage time, primary activities on social media, and primary usage purpose.

At the end of the intervention, additional assessment questions captured participants' perceived effects of the treatment and usability feedback. An open-ended question was also available for participants to share opinions or concerns. For the control group, before the beginning of intervention, open-ended questions about relevant events and mental health treatment initiation during waiting period were added. Reminder calls also provided feedback on pre- and post-measurements and clarified intervention procedures.

Participant engagement with the intervention was tracked through a platform feature that enabled clinicians to monitor the number of modules and exercises accessed and total logins. Feedback could also be submitted by participants at the end of each module and post-treatment assessment.

### 3.3 Statistical analysis

Descriptive analyses, including *t*-tests and χ^2^ tests, were conducted to assess baseline characteristics as well as differences between completers and non-completers.

A significance level of *p* < 0.05 was used, and effect sizes were calculated using Cohen's *d* to compare outcomes within and between group effects. Effect sizes were interpreted as small (*d* = 0.20), medium (*d* = 0.50), and large (*d* = 0.80) (Cohen, 1988).

Since the dropout rate was 40 % (36/92), the results of complete-case analysis (CCA) were considered (Jakobsen et al., 2017). However, we also used Intention-to-Treat Analysis (ITT) using Multiple Imputations method to handle the missing data, to check if any difference would be found. Missing data were handled with 20 imputations, following recommendations by Enders (2017). The impact of the treatment on outcome variables was analyzed using Multiple Regression Analysis with simultaneous entry, where all predictors were entered into the regression model simultaneously. Predictor variables included group condition and pre-treatment measures, while post-treatment measures served as the outcome variable. All analyses were conducted using IBM SPSS Statistics (version 27).

## 4 Results

### 4.1 Sociodemographics characteristics

The sociodemographic characteristics of the sample are summarized in Table 2. The majority of participants were female (82.6 %) and students (85.9 %), with a mean age of 21.36 years (*SD* = 1.84). Regarding psychological diagnoses, three participants initially reported substance use; however, subsequent interviews confirmed they did not meet the criteria for a substance use disorder. Among participants taking prescribed medications (*n* = 13), none reported dosage changes prior to the intervention.

**Table 2:** Sociodemographics characteristics of included participants.

	Treatment Group (*n* = 46)		Control Group (n = 46)		Total (*n* = 92)	
	n	%	n	%	n	%
Gender						
Female	38	(82.6)	38	(82.6)	76	(82.6)
Male	7	(15.2)	6	(13.0)	13	(14.1)
Other	1	(2.2)	2	(4.3)	3	(3.3)

Age						
Mean (SD)	21.63	(1.74)	21.09	(1.91)	21.36	(1.84)
Min – Max	18	(24)	18	(24)	18	(24)

Education level						
Complete high school / Higher education incomplete	37	(80.4)	39	(84.8)	76	(82.6)
Higher education complete	4	(8.7)	4	(8.7)	8	(8.7)
Postgraduate incomplete	4	(8.7)	3	(6.5)	7	(7.6)
Postgraduate complete	1	(2.2)	0	(0)	1	(1.1)

Civil state						
Single	42	(91.3)	44	(95.7)	86	(93.5)
Married	1	(2.2)	0	(0)	1	(1.1)
Stable union	2	(4.3)	2	(4.3)	4	(4.3)
Divorced	1	(2.2)	0	(0)	1	(1.1)

Student						
Yes	38	(82.6)	41	(89.1)	79	(85.9)
No	8	(17.4)	5	(10.9)	13	(14.1)

Working with a salary						
Yes	13	(28.3)	16	(34.8)	29	(31.5)
No	33	(71.7)	30	(65.2)	63	(68.5)

Psychological diagnostic (n = 3)						
Bipolar	0	(0)	0	(0)	0	(0)
Schizophrenia	0	(0)	0	(0)	0	(0)
Substance abuse	1	(2.2)	2	(4.3)	3	(100)

Psychological or psychiatric treatment						
Yes	5	(10.9)	2	(4.3)	7	(7.6)
No	41	(89.1)	44	(95.7)	85	(92.4)

Psychopharmacological medication						
Yes	6	(13.0)	7	(15.2)	13	(14.1)
No	40	(87.0)	39	(84.8)	79	(85.9)

Psychopharmacological medication changes in the last 3 months (n = 13)						
Yes	0	(0)	0	(0)	0	(0)
No	6	(46.15)	7	(53.85)	13	(100)

Housing						
Lives alone	6	(13.0)	3	(6.5)	9	(9.8)
Lives with family members/partner	23	(50)	31	(67.4)	54	(58.6)
Lives with other people (student republic, accommodation, etc.)	16	(34.8)	11	(23.9)	27	(29.4)
Lives in different accommodations (hotel, boarding house, etc.)	1	(2.2)	1	(2.2)	2	(2.2)

Monthly income (BRL)						
up to R$ 1.650.00	11	(23.9)	12	(26.1)	23	(25.0)
R$ 1.650.01 to R$ 3.300.00	19	(41.3)	19	(41.3)	38	(41.3)
R$ 3.300.01 to R$ 4.950.00	5	(10.9)	10	(21.7)	15	(16.3)
R$ 4.950.01 to R$ 6.600.00)	3	(6.5)	1	(2.2)	4	(4.3)
R$ 6.600.01 to R$ 11.000.00	4	(8.7)	3	(6.5)	7	(7.6)
R$ 11.000.01 to R$ 33.000.00	4	(8.7)	1	(2.2)	5	(5.4)

No statistically significant differences were found between groups regarding demographic characteristics or baseline symptomatology, as confirmed by independent *t*-tests and χ^2^-tests (*p* > 0.05), indicating that groups were equivalent at the outset of the intervention period.

### 4.2 Treatment dropout, adherence, and missing data

Attrition was considered low, with only one participant per group discontinuing treatment during the ongoing intervention. Consequently, at T1 45 participants were enrolled in the treatment group and 31 in the control group at T2. Participants who did not complete the post-treatment assessment were considered dropouts, while those who finished were defined as completers. The overall assessment dropout rate at post-treatment T2 was 40 % (*n* = 34), with 48 % (*n* = 21) in the treatment group and 30 % (*n* = 13) in the control group. No significant differences between completers and non-completers were observed in pretreatment symptoms using *t*-tests or *χ*^2^ tests for sociodemographic characteristics (*p* > 0.05), except for the “Where do you live?” question (χ^2^ = 18.2; df = 5; *p* = 0.003).

Adherence was defined as participants opening at least half of the modules (4 out of 8). More than half of the treatment group (55.5 %, *n* = 25) adhered to treatment, and 76 % (*n* = 19) of those opened all 8 modules. Participants in the treatment group (*n* = 45) opened, on average, 4.58 modules (SD = 3.13), with significant difference was found between those who completed post-test (*n* = 24, *M* = 7.16 modules, *SD* = 1.92) and assessment dropouts (*n* = 25, *M* = 4.4 modules, *SD* = 3.14) (χ^2^ = 28.2; df = 1; *p* < 0.001). On average, across all conditions (*n* = 76), participants opened 4.57 modules (*SD* = 3.12). No significant correlations with any outcome measures were found in relation to treatment adherence (*p* > 0.05) or modules completion (*p* > 0.05), except for SAS-SV (*r* = −0.439, *p* = 0.032) and the number of modules opened. Table 1 presents the ranking and percentage of each module access.

During intervention, most logins were made through mobile phone (*n* = 338), followed by desktop (*n* = 291) and tablets (*n* = 10). Four participants never logged into the platform. A total of 76 messages were sent by the treatment group participants (*n* = 45) (*M* = 1.64, *SD* = 2.75), with 65 messages sent by completers group (*M* = 2.7, *SD* = 3.39). Regarding time spent working with participants, therapists were instructed to spend a maximum of 15 min per participant per week, including feedback and support on demand. The exact time was not calculated, but during discussions, no case that required longer periods was reported.

### 4.3 Analysis of treatment effects

Table 3 shows the descriptives statistics for pre and post-measures for both groups. Table 4 shows the regression coefficients when group condition was used as a predictor for each measure in post-treatment. For the primary outcomes, the regression model showed a significant unstandardized regression coefficient for group condition, with b = −5.757 95 % CI [−9.629, −1.885], *t =* −2.983, *p* = 0.004 for anxiety symptoms and b = −7.605 95 % CI [−11.972, −3.237], *t* = −3492, *p* = 0.001 for depression symptoms. The effect size was large for depression (*d* = 0.87) and moderate to anxiety (*d* = 0.75) symptoms.

**Table 3:** Means and SDs for each measure divided by condition and assessment.

Measure	Assessment	Treatment			Control		
		mean	SD	n	mean	SD	n
DASS-21 - Anxiety	Pre	17.61	9.64	46	16.13	8.26	46
	Post	9.00	5.93	24	14.94	9.17	32
DASS-21 - Depression	Pre	20.91	11.24	46	19.17	9.46	46
	Post	10.08	6.76	24	18	10.56	32
DASS-21 - Stress	Pre	23.61	7.98	46	24.48	7.54	46
	Post	15.67	4.44	24	23.69	7.17	32
ISI	Pre	11.85	5.73	46	11.28	5.11	46
	Post	7.25	4.98	24	12.34	6.29	32
APSR	Pre	122.41	15.73	46	117.54	18.5	46
	Post	114.79	18.72	24	113.62	15.23	32
SAS	Pre	37.59	10.4	46	32.74	8.4	46
	Post	32.79	9.51	24	32.28	8.66	32
BBQ	Pre	38.61	19.2	46	39.65	18.98	46
	Post	50	16.5	24	38.69	16.01	32
SMDS	Pre	3.72	1.82	46	3.2	1.63	46
	Post	3.46	1.77	24	3.13	1.947	32

DASS-21. Depression. Anxiety and Stress Scale – 21 items; ISI. Insomnia Severity Index; APS-R. Almost Perfect Scale – Revised; BBQ. Brunnsviken Brief Quality of Life Scale; SAS-SV. Smartphone Addiction Scale-Short Version; SMDS-SF. Social Media Disorder Scale-Short Form.

**Table 4:** Regression model of the impact of group condition on primary and secondary outcome measures with variance explained by pre-treatment measure included.

Measure	Unstandardized coefficients		Standardized coefficients			Between-group effect size [95 % CI]
	B [95 % CI]	SE B	β	t	p	
DASS-21 - Anxiety	−5.757 [−9.629, −1.885]	1.930	−0.341	−2.983	0.004*	0.75 [0.186, 1.31]
DASS-21 - Depression	−7.605 [−11.972, −3.237]	2.178	−0.384	−3.492	0.001*	0.87 [0.3, 1.43]
DASS-21 - Stress	−7.824 [−10.999, −4.649]	1.583	−0.535	−4.942	0.000*	1.3 [0.706, 1.9]
ISI	−4.557 [−6.884, −2.229]	1.161	−0.364	−3.926	0.000*	0.88 [0.315, 1.45]
APSR	−4.964 [−11.812, 1.885]	3.414	−0.149	−1.454	0.152	−0.07 [−0.611, 0.472]
SAS	−2.419 [−6.037, 1.198]	1.804	−0.135	−1.341	0.186	−0.06 [−0.598, 0.485]
BBQ	11.786 [3.951, 19.620]	3.906	0.345	3.017	0.004*	−0.7 [−1.23, 0.14]
SMDS	−0.076 [−0.837, 0.685]	0.379	−0.020	−0.200	0.842	−0.18 [−0.72, 0.365]

For secondary measures, group condition also showed a significant effect as a predictor of post-treatment measures for stress [b = −7.824, 95 % CI [−10.999,−4.649], *t.*

= −4.942, *p* = 0.000], insomnia [b = −4.557, 95 % CI [−6.884,−2.229], *t* = −3.926, *p* = 0.000], and quality of life [b = 11.786, 95 % CI [3.951,19.620], *t* = 3.017, *p* = 0.004].

The effect size was large for stress (*d* = 1.3) and insomnia (*d* = 0.88), and moderate for quality of life (*d* = −0.7).

Since the study had a 40 % assessment dropout rate, the results presented here were obtained using observed data from the complete case analysis (CCA). However, an intention-to-treat (ITT) analysis was also conducted and showed no significant differences. Analysis are available as supplementary material.

### 4.4 Reliable change and negative effects

The Reliable Change Index (RCI) was calculated using Jacobson and Truax (1991) equation for anxiety and depression symptoms - the primary outcomes that showed significant changes between pre- and post-treatment. Utilizing the test-retest reliability for the DASS-21 as reported by (Silva et al., 2016), an RCI of 5.48 was determined for anxiety, and 5.71 for depression.

In the treatment group (*n* = 24), 37.5 % (*n* = 9) exhibited a reliable change in anxiety symptoms, while 29.2 % (*n* = 7) demonstrated a reliable change in depressive symptoms, as shown in Table 5. χ^2^ tests revealed significant differences between the control and treatment groups, with the deterioration percentages displaying the most pronounced differences.

**Table 5:** χ^2^ tests of reliable change and deterioration in post-treatment for both groups.

Measure	Treatment (n = 24)			Control (*n* = 32)		
	Change %(n)	Unchanged %(n)	Deterioration %(n)	Change %(n)	Unchanged %(n)	Deterioration %(n)
DASS-21 - Anxiety	37.5 (9)	45.8 (11)	16.7 (4)	12.5 (4)	46.9 (15)	40.6 (13)
	χ2 = 6.29; df = 2; *p* = 0.043					
DASS-21 - Depression	29.2 (7)	62.5 (15)	8.3 (2)	12.5 (4)	40.6 (13)	46.9 (15)
	χ2 = 9.96; df = 2; *p* = 0.007					

### 4.5 Follow-up

All measures were reassessed six months after the treatment group conclusion. Fifteen participants in the treatment group responded to the follow-up. For this investigation, only the measures that showed significant changes in post-treatment were evaluated in the follow-up - anxiety, depression, stress, insomnia, and quality of life. Paired samples *t*-tests were utilized to analyze these data. No significant differences were found between post-test and follow-up test results (all *p's* > 0.05). Table 6 summarizes the findings and shows effect sizes and confidence intervals.

**Table 6:** Paired *t*-tests of differences between post-treatment and 6 month follow-up for treatment group (*n* = 15).

Measure	Post	Follow-up
	M (SD)	M (SD)
DASS - Anxiety	7.73 (6.23)	10.27 (8.65)
	t(14) = −1.258; *p* = 0.229; *d* = −0.325, 95 % CI [−0.839, 0.201]	
DASS - Depression	10.4 (7.57)	12 (13.22)
	t(14) = −0.494; *p* = 0.629; *d* = −0.128, 95 % CI [−0.634, 0.383]	
DASS - Stress	14.67 (4.76)	15.87 (10.46)
	t(14) = −0.532; *p* = 0.603; *d* = −0.137, 95 % CI [−0.643, 0.374]	
ISI	5.6 (4.93)	6.67 (5.22)
	t(14) = −0.897; *p* = 0.385; *d* = −0.232, 95 % CI [−0.741, 0.286]	
BBQ	49.47 (17.78)	46.93 (22.36)
	t(14) = 0.596; *p* = 0.561; *d* = 0.154, 95 % CI [−0.358, 0.660]	

## 5 Discussion

This pilot study investigated the effects of a tailored Internet-based Cognitive Behavioral Therapy (ICBT) intervention in a young adult population to reduce symptoms of anxiety and depression. Secondary outcomes included stress, insomnia, perfectionism, quality of life, and problematic internet use. The results demonstrated that the intervention was effective in reducing anxiety, depression, stress, and insomnia symptoms, while also improving perceived quality of life. It is important to note that since analyses were conducted using complete case analysis, a more liberal approach that should be considered when interpreting these results. These findings suggest that such an intervention could be not only be feasible within the Brazilian context but also potentially impactful for addressing mental health challenges in this demographic.

Regarding symptom reduction, the DASS-21 revealed a large effect on depression (d = 0.87) and stress symptoms (d = 1.3) and a moderate effect on anxiety symptoms (d = 0.75). Additionally, the ISI showed a large effect on insomnia symptoms.

(d = 0.88), while the BBQ indicated moderate improvements in quality of life (d = −0.7).

However, no statistically significant effects were found on the APS-R (d = −0.07), SASSV (d = −0.06), or SMDS-SF (d = −0.18). It is worth noting that the confidence intervals found in this study were wide, ranging from small to very large. Therefore, the findings and any conclusions about clinical significance should be interpreted with caution.

No significant differences were observed between the post-treatment outcomes and the six-month follow-up, which could suggest that the intervention's effects were maintained over time. However, since only 15 participants completed the 6-month follow-up (32.6 %) the interpretability of long-term effects is limited in this study.

These findings align with previous studies demonstrating the effectiveness of tailored ICBT interventions with therapist support via text messages for young people (Berg et al., 2022; Topooco et al., 2018). The findings also concur with research demonstrating significant changes in quality of life following asynchronous message based ICBT (Aminoff et al., 2023; Berg et al., 2022; Gasslander et al., 2022; Käll et al., 2020; Lindhe et al., 2023). Additionally, 37.5 % of participants in the treatment group exhibited reliable improvement in anxiety symptoms, and 29.7 % showed reliable improvement in depressive symptoms, according to the Reliable Change Index (RCI).

The intervention also appeared to prevent symptom deterioration, as the control group experienced significantly higher deterioration during the waitlist period. Although further research is needed to explore this data, these findings suggest that such interventions may serve as valuable tools within the public health system, particularly as an accessible and low-intensity option while individuals await more intensive care (Amaral et al., 2018).

The intervention modules focused on behavioral activation and exposure as primary CBT techniques to address common mental health symptoms, complemented by cognitive restructuring, emotional regulation, and the identification of maladaptive patterns. These techniques were adapted and presented through tailored modules, supporting the notion that personalized programs may enhance effectiveness (Aminoff et al., 2023; Andersson et al., 2019a; Gasslander et al., 2022; Käll et al., 2020;

Lindhe et al., 2023; Păsărelu et al., 2017). Considering associated risk factors with common mental health symptoms, such as problematic internet use, insomnia, and low self-esteem (Ramón-Arbués et al., 2020), modules addressing these issues may have contributed to the observed outcomes.

The overall assessment dropout rate at post-treatment was 40 % (*n* = 34), with 48 % (*n* = 21) in the treatment group and 30 % (*n* = 13) in the control group. While 55.5 % of participants in the treatment group adhered to the intervention, completing an average of 4.58 modules (SD = 3.13), dropout rates were relatively high compared to similar interventions (Berg et al., 2022; Topooco et al., 2018). However, these rates are consistent with findings from other Brazilian studies on ICBT (Lopes et al., 2023).

We hypothesize that the high dropout rate may be partly due to the novelty of self-guided ICBT in Brazil, which may not yet be fully accepted or well understood or widely accepted by the population. Additionally, given the systemic challenges within Brazil's healthcare system, participants with more severe symptoms or greater needs may have felt that the low-intensity intervention was insufficient, leading to higher dropout rates. Further investigation is required to identify and address the factors contributing to attrition in this context.

Another notable finding was the high prevalence of mobile device use for accessing the intervention, consistent with national data indicating that the majority of Internet users in Brazil access online content via mobile phones (CGI, 2020). This highlights the feasibility of delivering ICBT programs via mobile platforms, particularly for lower socioeconomic groups who primarily use smartphones for internet access. Moreover, the average time required per participant, aligning with previous research (Aminoff et al., 2023; Berg et al., 2022; Käll et al., 2020; Lindhe et al., 2023) indicating that <30 min per week of therapist time is sufficient in such interventions — underscoring the low-resource nature of this intervention, making it a viable option for Brazil's public health system.

Defining adherence poses a challenge in this and similar studies, and it may be a fourth limitation. In our study, adherence was defined as completing half or more of the program. Significant results were observed in the treatment group even when participants completed around half of the modules, even if there appears to be no dose response relationships in prior research (Aminoff et al., 2023; Berg et al., 2022; Käll et al., 2020; Topooco et al., 2018). However, different studies employ varying definitions of adherence.

While the findings suggest that the intervention may help reduce symptoms of anxiety and depression, some limitations should be considered when interpreting these results. Regarding methodology, the use of a transdiagnostic and tailored intervention makes it unclear which components of the program drove the observed effects. Additionally, the use of a waitlist control condition also makes it challenging to isolate nonspecific treatment effects (Patterson et al., 2016). The recruitment strategy, which relied on social media and university networks, likely introduced selection bias by attracting individuals who were actively seeking help online. While this reflects real-world patterns of ICBT engagement, it may have reduced the representativeness of the sample.

The sample's sociodemographic composition — primarily female university students — further limits generalization. Although these characteristics are consistent with previous ICBT studies (Andersson, 2024), caution should be exercised when applying these findings to wider populations or public health settings. Also, high assessment dropout rates (40 %) and during follow-up (32.6 %) also impact the robustness of the findings. Although multiple imputation analyses did not reveal significant differences between completers and non-completers, the lack of intent-to-treat analyses limits the certainty with which we can interpret the treatment's effectiveness. Moreover, the reasons for dropout remain unknown, since those participants didn't want to be contacted during post-assessments and follow-up. Those limitations highlight the need for future studies to include more detailed clinical assessments and potential strategies to enhance adherence beyond the ones used in this study, such as gamification, for example (Sardi et al., 2017).

Lastly, while ICBT shows potential for addressing mental health needs in low- and middle-income countries like Brazil, its broader implementation still faces important barriers, such as infrastructure and user engagement. Although this was not the goal of the present study, discussion of digital mental health tools must go beyond efficacy and consider real-world challenges such as accessibility, scalability, and sustainability. Recent frameworks like TEQUILA (Löchner et al., 2025) —Trust, Evidence, Quality, Usability, Interest, Liability, Accreditation— offer a comprehensive lens for evaluating digital interventions, aiming to ensure efficacy, safety, and user confidence. Future research should align more closely with frameworks like TEQUILA to ensure that digital mental health innovations are not only effective but also trustworthy and sustainable in the long term.

## 6 Conclusions

This pilot RCT provides preliminary evidence that a tailored ICBT intervention could be a feasible and effective approach for reducing anxiety and depressive symptoms among young Brazilians. The intervention not only improved primary outcomes but also showed improvements in secondary outcomes, such as stress, insomnia, and quality of life, demonstrating its potential to address common mental health issues in this population. From a public health perspective, ICBT represents a cost-effective and accessible option for reducing mental health disparities in countries like Brazil. However, since this is a pilot trial, those preliminary results should be taken with caution, considering some limitations of this study. Further research with larger samples and diverse populations is warranted to confirm these findings, explore long-term effects and refine implementation strategies to maximize the intervention's impact.

## CRediT authorship contribution statement

JM is the principal author and was responsible for writing the original draft, project administration, editing and funding acquisition.

VA helped with statistical analyses and advised on treatment implementation.

CBN collaborated with writing review and supervision of this manuscript.

GA was responsible for conceptualization, supervision, reviewing and editing writing.

## Declaration of Generative AI and AI-assisted technologies in the writing process

During the preparation of this work the author(s) used Copilot and ChatGPT in order to improve readability and language of the manuscript. After using this tool/service, the author(s) reviewed and edited the content as needed and take(s) full responsibility for the content of the published article.

## Funding

This work was supported by the 10.13039/501100003593National Council for Scientific and Technological Development (CNPq); 10.13039/501100002322Coordination for the Improvement of Higher Education Personnel (CAPES) and The 10.13039/501100001807São Paulo Research Foundation (FAPESP).

## Declaration of competing interest

The authors declare that they have no known competing financial interests or personal relationships that could have appeared to influence the work reported in this paper.

## Appendix A Supplementary data

Supplementary material Image 1
