# Supplementary material for: Internet-based cognitive behavioral therapy for anxiety and depressive symptoms in Brazilian emerging adults: A pilot randomized controlled trial
Source: Internet Interv. 2025 Jul 9;41:100854. doi: 10.1016/j.invent.2025.100854 (PMC12274321; doi:10.1016/j.invent.2025.100854)
Supplement: Supplementary file 1 — Supplementary material [file mmc1.docx]

| **Measure** | **Unstandardized coefficients** | | **Standardized coefficients** | | | **Between-group effect size [95% CI]** |
| --- | --- | --- | --- | --- | --- | --- |
|  | **B [95 % CI]** | **SE B** | **β** | **t** | **p** |  |
| DASS-21 - Anxiety | -4,682 [-8,295, -1,070] | 1,841 | -0,286 | -2,544 | 0,011 | 0,64 [0.22, 1.05] |
| DASS-21 - Depression | -5,963 [-9,991, -1,935] | 2,054 | -0,315 | -2,904 | 0,004 | 0,72 [0.3, 1.15] |
| DASS-21 - Stress | -6,089[-9,270, -2,908] | 1,621 | -0,417 | -3,756 | 0,000 | 0,9 [0.56, 1.43] |
| ISI | -4,473 [-6,740, -2,206] | 1,155 | -0,349 | -3,871 | 0,000 | 0,79 [0.37, 1.22] |
| APSR | -2,795 [-10,366, 4,776] | 3,845 | -0,078 | -0,727 | 0,468 | 0,15 [-0.25, 0.56] |
| SAS | -2,359 [-6,389, 1,671] | 2,052 | -0,121 | -1,15 | 0,251 | 0,24 [-0.16, 0.65] |
| BBQ | 10,066 [1,433, 18,699] | 4,386 | 0,275 | 2,295 | 0,022 | -0,58 [-1, -0.16] |
| SMDS | -0,192 [-0,957, 0,572] | 0,39 | -0,047 | -0,493 | 0,622 | 0,1 [-0.3, 0.51] |

Table 1 - Regression model of the impact of group condition on primary and secondary outcome measures with variance explained by pre-treatment measure included with imputed data

DASS-21, Depression, Anxiety and Stress Scale – 21 items; ISI, Insomnia Severity Index; APS-R, Almost Perfect Scale – Revised; BBQ, Brunnsviken Brief Quality of Life Scale; SAS-SV, Smartphone Addiction Scale-Short Version; SMDS-SF, Social Media Disorder Scale-Short Form.

Table 2 - Pooled means, SDs, and the number of participants with imputed data for each measure divided by condition and assessment point

| **Measure** | **Assessment point** | **Treatment** | | | **Control** | | |
| --- | --- | --- | --- | --- | --- | --- | --- |
|  |  | **mean** | **SD** | **n** | **mean** | **SD** | **n** |
| DASS-21 - Anxiety | Pre | 13,82 | 9.64 | 46 | 16,13 | 8,26 | 46 |
|  | Post | 9,14 | 5,94 | 46 | 13,82 | 8,43 | 46 |
| DASS-21 - Depression | Pre | 20,91 | 11,24 | 46 | 19,17 | 9,46 | 46 |
|  | Post | 10,21 | 6,07 | 46 | 16,17 | 9,77 | 46 |
| DASS-21 - Stress | Pre | 23,61 | 7,98 | 46 | 24,48 | 7,54 | 46 |
|  | Post | 15,63 | 4,42 | 46 | 21,72 | 7,35 | 46 |
| ISI | Pre | 11,85 | 5,73 | 46 | 11,28 | 5,11 | 46 |
|  | Post | 7,68 | 4,94 | 46 | 12,15 | 6,16 | 46 |
| APSR | Pre | 122,41 | 15,73 | 46 | 117,54 | 18,5 | 46 |
|  | Post | 111,80 | 19,08 | 46 | 114,6 | 15,96 | 46 |
| SAS | Pre | 37,59 | 10,4 | 46 | 32,74 | 8,4 | 46 |
|  | Post | 31,35 | 9,68 | 46 | 33,68 | 9,11 | 46 |
| BBQ | Pre | 38,61 | 19,2 | 46 | 39,65 | 18,98 | 46 |
|  | Post | 50,94 | 17,11 | 46 | 40,88 | 17,04 | 46 |
| SMDS | Pre | 3,72 | 1,82 | 46 | 3,2 | 1,63 | 46 |
|  | Post | 3,26 | 1,79 | 46 | 3,46 | 1,99 | 46 |
